# Continued alcohol consumption and hepatic encephalopathy determine quality of life and psychosocial burden of caregivers in patients with liver cirrhosis

**DOI:** 10.1186/s12955-022-01923-z

**Published:** 2022-02-08

**Authors:** Michael Nagel, Vanessa Weidner, Sina Schulz, Jens U. Marquardt, Peter R. Galle, Jörn M. Schattenberg, Marc Nguyen-Tat, Marcus-Alexander Wörns, Christian Labenz

**Affiliations:** 1 Department of Internal Medicine I, University Medical Center Mainz of the Johannes Gutenberg-University Mainz, Mainz, Germany; 2 Cirrhosis Center Mainz (CCM), University Medical Center of the Johannes Gutenberg-University Mainz, Mainz, Germany; 3 First Department of Medicine, University Hospital Schleswig-Holstein, Campus Lübeck, Lübeck, Germany; 4 Metabolic Liver Research Program, University Medical Center of the Johannes Gutenberg-University Mainz, Mainz, Germany; 5 Medical Center Kempten, Department of Gastroenterology, Kempten, Germany; 6 Department of Gastroenterology, Hematology, Oncology, and Endocrinology, Klinikum Dortmund, Beurhausstraße 40, 44137 Dortmund, Germany

**Keywords:** Quality of life, Complications of liver cirrhosis, Decompensated liver cirrhosis, Burden of disease

## Abstract

**Background:**

Patients with liver cirrhosis suffer from significantly reduced health-related quality of life and are often dependent on support from caregivers. In this context, caregivers often suffer from impaired quality of life (QoL) as well as psychosocial burden (PB). The aim of the present study was to identify factors influencing QoL and PB of caregivers in order to improve the social care of patients and caregivers.

**Methods:**

In this cross-sectional study, 106 patients with liver cirrhosis and their caregivers were included. (Health-related) QoL was surveyed in patients (CLDQ) and caregivers (SF-36) and PB was determined by Zarit Burden Interview.

**Results:**

Alcohol related liver cirrhosis (55%) was the predominant etiology of liver cirrhosis and the median MELD of the cohort was 14. QoL did not differ between patients with and without alcohol-related liver cirrhosis (*p* = 0.6). In multivariable analysis, continued alcohol consumption (*p* = 0.020), a history of hepatic encephalopathy (HE) (*p* = 0.010), poorer QoL of patients (*p* = 0.030) and poorer QoL of caregivers (*p* = 0.005) were associated with a higher PB of caregivers. Factors independently associated with poorer QoL of caregivers were continued alcohol consumption (*p* = 0.003) and a higher PB of caregivers (*p* = 0.030).

**Conclusion:**

Caregivers of patients with liver cirrhosis suffer from impaired QoL and PB, especially in case of continued alcohol consumption or the occurrence of HE.

**Supplementary Information:**

The online version contains supplementary material available at 10.1186/s12955-022-01923-z.

## Introduction

Chronic liver diseases are among the most common diseases worldwide. Liver cirrhosis is the end-stage of almost all chronic liver diseases and represents a huge burden for affected patients with high morbidity and mortality. While the prognosis of patients with compensated liver cirrhosis is fairly good, there is an excess in the mortality rate when decompensation of liver cirrhosis (e.g. ascites, gastrointestinal bleeding, hepatorenal syndrome, or hepatic encephalopathy (HE) occurs [1]. Besides the detrimental effect of these complications on the respective patient’s prognosis, health-related quality of life (HRQoL) deteriorates when decompensation of liver cirrhosis occurs [2]. Additionally, a recent study demonstrated that poorer HRQoL is an indicator of poor prognosis in patients with liver cirrhosis and ascites in the long term [3]. While there are plenty of studies investigating the impact of different complications of liver cirrhosis on the patient’s HRQoL [4–6], data on the impact of complications of liver cirrhosis on patients’ caregivers are currently scarce. In a smaller precursor study conducted at our center, we were able to identify a detrimental effect of acute-on-chronic liver failure (ACLF) on the psychosocial burden (PB) of caregivers [5]. Caregivers are of pivotal importance in the management of patients with decompensated liver cirrhosis. In the outpatient setting, most patients with liver cirrhosis need continued support to plan their medication and caregivers might be helpful in the detection of early signs of liver decompensation. Therefore, this social support is of considerable importance in the management of patients with liver cirrhosis. Due to this necessity of a close bond between patients and caregivers, it seems reasonable that the occurrence of complications of liver cirrhosis might impact caregivers’ quality of life (QoL) and PB. In line with this hypothesis, a study by Fabrellas et al. identified a profound psychological impact of HE on patients’ as well as on their caregivers’ QoL [7]. Additionally, a recent study indicated that the burden on caregivers is particularly pronounced in patients with ongoing alcohol abuse [8]. However, data on the impact of other complications of liver cirrhosis on informal caregivers are still limited. Therefore, the aim of the present study was to investigate the influence of HRQoL of patients and the presence/occurrence of complications of liver cirrhosis on the QoL and PB of caregivers.

## Methods

### Patients and caregivers

**Fig. 1:**
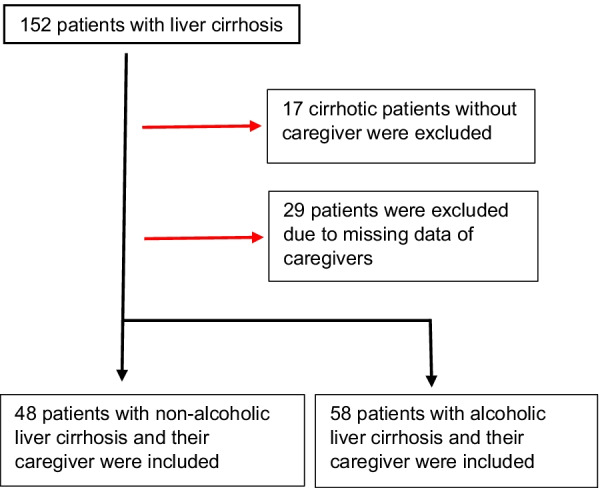
Flow diagram showing the reasons for dropout of patients

In total, 152 in- and outpatients with liver cirrhosis were prospectively recruited for this study between May 2017 and May 2018 at the Cirrhosis Center Mainz (CCM) at the University Medical Center of the Johannes Gutenberg-University in Mainz, Germany. A patient flow chart is given in Fig. 1. Patients with active malignancies were not approached for this study. Diagnosis of liver cirrhosis was established by typical appearance on ultrasound, computed tomography/magnetic resonance imaging or by liver biopsy. At presentation or inpatient admission, all patients received a standardized medical history and a laboratory examination. In addition to general epidemiological data such as age and gender, etiology of liver cirrhosis was determined and liver function was assessed by Model of End-stage Liver Disease (MELD) and Child–Pugh (CP) score [9, 10]. Caregivers were asked regarding their personal relationship to the patient. Data not only on patients’ HRQoL, but also caregivers’ QoL and PB have been published in this previous study focusing on the impact of ACLF on these measures [5].

### Assessment of QoL and PB in patients with liver cirrhosis and their caregivers

To assess HRQoL in patients with liver cirrhosis, the validated German version of the Chronic Liver Disease Questionnaire (CLDQ) was used [11]. The questionnaire contains 29 items, which can be grouped into the domains activity, fatigue, worries, abdominal symptoms, and systemic symptoms. Each category can be assessed separately between groups. Higher results indicate better QoL. The results of the CLDQ score are presented on a seven-point Likertscale.

To assess QoL and PB, caregivers were asked to complete the Short Form 36 Health Survey (SF-36) and the Zarit Burden Interview (ZBI) [12–14]. These two generic questionnaires were chosen because they are well validated to assess HRQoL and PB in healthy individuals. The SF-36 contains 36 questions which can be grouped into physical strength, social strength, environmental strength, and mental strength. Each category can be assessed separately between groups. The lower the score the greater the perceived disability, i.e., a score of zero is equivalent to maximum disability and a score of 100 is equivalent to no disability [13, 15]. The ZBI contains a total of 22 questions, which were answered by the caregivers on a 5-point Likert-scale. Responses were finally used to derive the ZBI total score (range 0 (no burden) to 88 (highest burden)). Interpretation of the ZBI was done as previously described [12, 16]. Briefly, a low PB was assumed for scores up to 20. A moderate or high PB was assumed in patients with scores between 21–40 or > 40, respectively.

### Ethics

The study was conducted according to the ethical guidelines of the 1975 Declaration of Helsinki and its later amendments. The ethics committee of the Landesärztekammer Rhineland-Palatine (Nr. 837.232.17 [11066]) approved the study protocol. Written informed consent was obtained from every participant.

### Statistical analysis

The statistical analyses were performed with IBM SPSS Statistic Version 23.0 (IBM Corp., Armonk, NY, USA). Quantitative data are expressed as medians with interquartile ranges (IQR).

The correlation of clinical und epidemiological factors with QoL and PB of caregivers was assessed by means of univariate analyses. Variables with a *p* < 0.1 in the univariate analysis were subsequently considered in a multivariate linear regression model for each score. To reliably identify factors being associated with SF-36 and ZBI, the final multivariate model was built based on a stepwise variable selection procedure for each score. Our complete data analysis is exploratory. Hence, no adjustments for multiple testing were performed. For all tests, we used a 0.05 level to define statistically relevant deviations from the respective null hypothesis. However, due to the large number of tests, *p* values should be interpreted with caution.

## Results

### Baseline characteristics of the study cohort

In total, 152 patients were screened for this study. 17 patients were excluded due to the unavailability of a respective caregiver. Of the remaining 135 patients, 29 were excluded due to missing data intended to be provided by the respective caregivers (ZBI and/or SF-36). Finally, a total of 106 patients were included in the analysis (Fig. 1).

In the total cohort, 58 (55%) patients had an alcohol-related liver cirrhosis. Median age of the total cohort was 63 (IQR 53; 69) years, and median MELD was 14 (IQR 10; 18). Median age of the caregivers was 59 (IQR 47; 66) years. Additional baseline characteristics of the total cohort are displayed in Table 1. For further analysis, the total cohort of all patients with liver cirrhosis and their caregivers was divided into alcoholic and non-alcoholic liver cirrhosis (Additional file 1: Table S1). Due to the different clinical therapy, the two cohorts were considered separately in order to identify specific influencing factors.

**Table 1:** Patient baseline characteristics at the time of study inclusion

Variable	Patients with liver cirrhosis (n = 106)
Male Gender n (%)	72 (68%)
Age of patients (years) median (IQR)	63 (53; 69)
Age of caregiver (years) median (IQR)	59 (47; 66)
Sodium (mmol/l) median (IQR)	138 (135; 140)
Creatinine (mg/dl) median (IQR)	0.9 (0.8; 1.4)
Bilirubin (mg/dl) median (IQR)	1.8 (1; 2.6)
Albumin (g/l) median (IQR)	31 (26; 34)
INR median (IQR)	1.4 (1.2; 1.6)
CRP (mg/l) median (IQR)	8.1 (3.5; 18.5)
White blood cell count (/nl) median (IQR)	5.7 (4.6; 7.6)
Hemoglobin (g/d) median (IQR)	12.3 (10.2; 13.6)
Platelets (/nl) median (IQR)	99 (72; 147)
MELD median (IQR)	14 (10; 18)
Child–Pugh score *A n (%)	49 (46%)
*B n (%)	41 (39%)
*C n (%)	16 (15%)
History of ascites n (%)	60 (57%)
History of spontaneous bacterial peritonitis n (%)	9 (8%)
History of hepatic encephalopathy n (%)	28 (26%)
History of variceal bleeding n (%)	21 (20%)
History of hepatorenal syndrome n (%)	11 (10%)

### Predictors of poorer QoL in caregivers

**Fig. 2:**
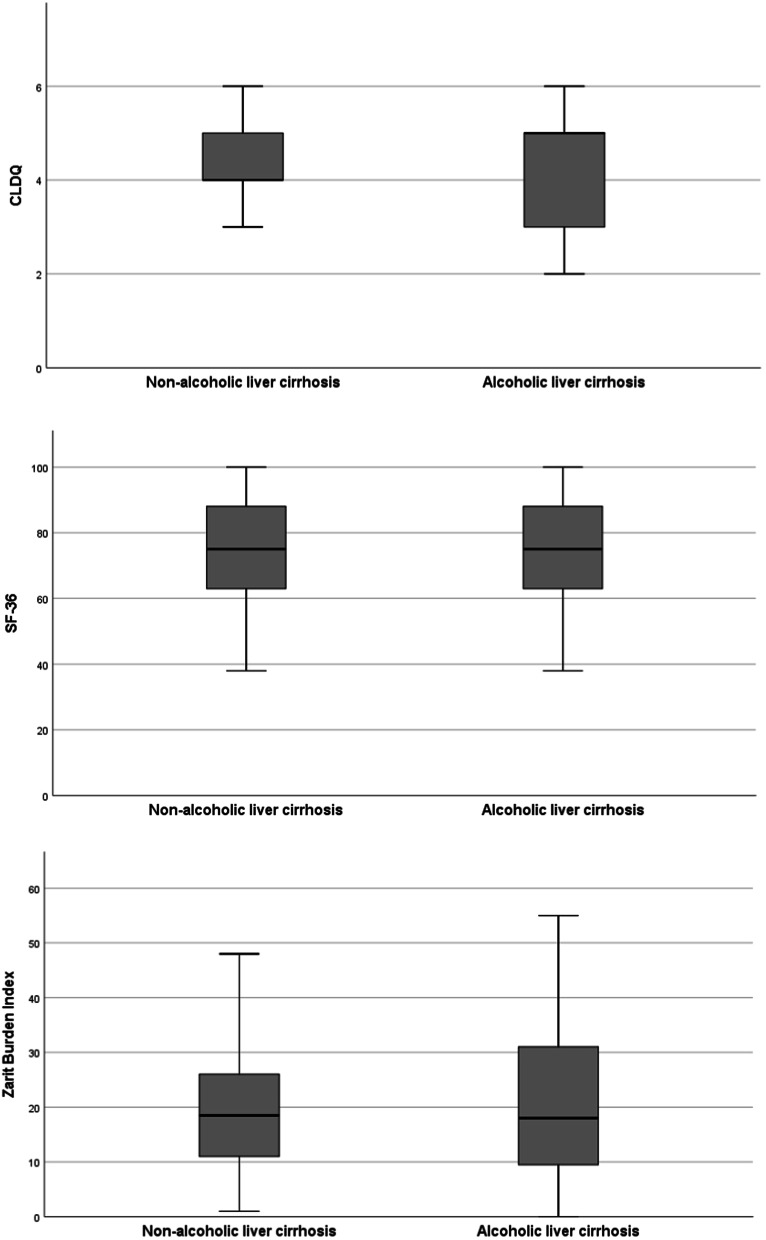
Health-related quality of life of patients with liver cirrhosis as well as quality of life and psychosocial burden of caregivers. **A** displays health-related quality of life (CLDQ) of patients with liver cirrhosis stratified by alcoholic and non-alcoholic etiology (not significant). **B** displays quality of life (SF-36) of caregivers stratified by alcoholic and non-alcoholic liver cirrhosis (not significant). **C** displays psychosocial burden (ZBI) of caregivers stratified by alcoholic and non-alcoholic liver cirrhosis (not significant)

To identify predictors of poorer QoL (SF-36) in caregivers of patients with liver cirrhosis, univariate and multivariate analyses were conducted (Table 2). In the total cohort, continued alcohol consumption of patients with liver cirrhosis (standardized β coefficient = 0.292, *p* = 0.003) and higher PB of caregiver (ZBI) (standardized β coefficient = − 0.310, *p* = 0.003) were the only independent factors associated with poorer QoL in caregivers in the multivariate linear regression analysis. To assess potential factors related to poorer QoL in caregivers of patients with alcoholic and non-alcoholic liver cirrhosis, we conducted separate subgroup analyses (Table 2). In patients with alcohol-related liver cirrhosis, continued alcohol consumption (standardized β coefficient = 0.355, *p* = 0.038) was the only independent factor associated with poorer QoL in caregivers in the multivariate linear regression analysis. In patients with non-alcoholic liver cirrhosis, a higher PB (ZBI) (standardized β coefficient = − 0.358, *p* = 0.010) remained the only independent predictor of poorer QoL in caregivers. The etiology of liver cirrhosis (alcoholic vs. non-alcoholic) had no impact on the QoL of caregivers (Fig. 2).

**Table 2:** Univariate and multivariate analyses to identify predictors for poorer quality of life (SF-36) of caregivers in the total cohort, in patients with alcoholic liver cirrhosis, and in patients with non-alcoholic liver cirrhosis

Variable	Univariate analysis		Multivariate analysis	
	r	*p* value	β	*p* value
Total cohort				
Continued alcohol consumption	0.259	0.079	0.292	0.003
Psychosocial burden of caregivers (ZBI)	− 0.275	0.005	− 0.310	0.003
Health-related Quality of life of patients (CLDQ)	0.182	0.062	0.139	0.167
Alcoholic liver cirrhosis				
Continued alcohol consumption	0.259	0.079	0.355	0.038
Psychosocial burden of caregivers (ZBI)	− 0.275	0.005	− 0.264	0.135
Health-related Quality of life of patient (CLDQ)	0.182	0.062	− 0.043	0.796
Non-alcoholic liver cirrhosis				
History of ascites	− 0.272	0.042	− 0.094	0.516
History of SBP	− 0.359	0.007	− 0.216	0.091
History of gastrointestinal bleeding	− 0.360	0.006	− 0.210	0.097
Health-related Quality of life of patients (CLDQ)	0.344	0.009	0.071	0.610
Psychosocial burden of caregivers (ZBI)	− 0.409	0.002	− 0.358	0.010

Gender 1 for male, 2 for female; 1 for Alcohol consumption, 0 for no alcohol consumption; 1 for history of hepatic encephalopathy, 0 for no history of hepatic encephalopathy. Factors not predictive for SF-36 in the univariate analysis were gender, age, age of caregiver, sodium, creatine, bilirubin, albumin, INR, CRP, leucocytes, hemoglobin, platelets, MELD, Child–Pugh status, history of hepatorenal syndrome. With the remaining factors, a multivariate linear regression model with inclusion variable selection was built

ZBI, Zarit burden interview; CLDQ, chronic liver disease questionnaire; SBP, spontaneous bacterial peritonitis; SF-36, Short Form Health 36; INR, international standardized ratio; CRP, C-reactive protein; MELD, model of end-stage liver disease

### Predictors of higher PB in caregivers

To identify predictors of higher PB (ZBI) in caregivers of patients with liver cirrhosis, univariate and multivariate analyses were conducted (Table 3). In the total cohort, continued alcohol consumption (standardized β coefficient = 0.280, *p* = 0.002), a history of HE (standardized β coefficient = 0.234, *p* = 0.010), poorer HRQoL of the patient (CLDQ) (standardized β coefficient = − 0.199, *p* = 0.030), and poorer QoL of caregiver (SF-36) (standardized β coefficient = − 0.250, *p* = 0.005) were independent factors associated with higher PB in caregivers. To assess potential factors related to higher PB (ZBI) in caregivers of patients with alcoholic and non-alcoholic liver cirrhosis, we conducted separate subgroup analyses (Table 3). In patients with alcoholic liver cirrhosis, continued alcohol consumption (standardized β coefficient = 0.305, *p* = 0.030) was the only independent factor associated with higher PB (ZBI) in caregivers in the multivariate linear regression analysis. In patients with non-alcoholic liver cirrhosis, poorer HRQoL of patients (CLDQ) (standardized β coefficient = 0.263, *p* = 0.039) and a history of or active HE (standardized β coefficient = 0.263, *p* = 0.039) remained the only independent predictors of higher PB in caregivers. The etiology of liver cirrhosis (alcoholic vs. non-alcoholic) had no impact on the PB of the caregivers (Fig. 2).

**Table 3:** Univariate and multivariate analysis to identify predictors for a higher psychosocial burden (PB) in caregivers in the total cohort, in patients with alcoholic liver cirrhosis, and in patients with non-alcoholic liver cirrhosis

Variable	Univariate analysis		Multivariate analysis	
	r	*p* value	β	*p* value
Total cohort				
Continued alcohol consumption	0.324	0.001	0.280	0.020
History of HE	0.390	< 0.001	0.234	0.010
Health-related Quality of life of patients (CLDQ)	− 0.419	< 0.001	− 0.199	0.030
Quality of life of caregivers (SF-36)	− 0.275	0.005	− 0.250	0.005
Alcoholic liver cirrhosis				
Quality of life of patients (CLDQ)	− 0.419	< 0.001	− 0.254	0.083
Continued calcohol consumption	0.449	0.002	0.305	0.030
History of HE	0.390	< 0.001	0.179	0.231
Non-alcoholic liver cirrhosis				
Health-related quality of life of patients (CLDQ)	− 0.4	0.002	0.263	0.039
Quality of life of caregivers (SF 36)	− 0.409	0.002	− 0.135	0.299
History of HE	0.389	0.003	0.263	0.039

Gender 1 for male, 2 for female; 1 for Alcohol consumption, 0 for no alcohol consumption; 1 for history of hepatic encephalopathy, 0 for no history of hepatic encephalopathy. Factors not predictive for ZBI in the univariate analysis were gender, age, age of caregiver, sodium, creatine, bilirubin, albumin, INR, CRP, leucocytes, hemoglobin, platelets, MELD, Child–Pugh status, history of ascites, history of spontaneously bacterial peritonitis, history of gastrointestinal bleeding, history of hepatorenal syndrome. With the remaining factors, a multivariate linear regression model with inclusion variable selection was built

HE, hepatic encephalopathy; SF-36, short form health 36; INR, international standardized ratio; CRP, C-reactive protein

## Discussion

In the present study, we identified potentially modifiable predictors regarding poorer QoL as well as higher PB in caregivers of patients with liver cirrhosis. We were able to demonstrate that continued alcohol consumption was associated with poorer QoL and a higher PB in caregivers, while an alcoholic etiology of liver cirrhosis per se had no impact on these factors. Additionally, we found that a history of HE represents a risk factor for higher PB in caregivers. Moreover, regarding a higher PB, caregivers’ QoL and patients’ HRQoL seem to influence each other in a negative sense.

Caregivers are of pivotal importance in the management of patients with (decompensated) liver cirrhosis. In the outpatient setting, most patients with liver cirrhosis need support to plan their medication. In addition, caregiver might be helpful in the detection of early signs of liver decompensation. Therefore, this social support is of utmost importance in the management of patients with liver cirrhosis. In this context, Rakoski et al. were able to demonstrate that patients with liver cirrhosis require on average 9 h of support per week from caregivers to cope with their disease [17]. However, data on the influence of disease-related factors on QoL and PB of caregivers, especially from Germany, are currently scarce. In our current study, we identified a history of or active HE as an independent predictor of higher PB. This association seemed to be especially true in patients with a non-alcoholic liver cirrhosis. This finding is in line with a recently published study by Fabrellas et al. Here, the authors identified a profound psychological impact of HE on patients as well as their caregivers, which also impairs QoL [7]. Another Italian study showed that PB of caregivers of patients with liver cirrhosis increases with the degree of HE [18]. The huge burden caused by an episode of overt HE is likely explained by the severe condition of patients at hospital admission (e.g. coma) and the aftermath of an episode. Patients with a history of overt HE often suffers from a residual cognitive deficit, which may affect daily living and could intensify the workload of a caregiver [19]. This hypothesis is strengthened by studies investigating other chronic neurological conditions. Here, chronic neurological diseases had a huge impact on QoL and PB of caregivers [20].

In our study, we identified continued alcohol consumption as a major determinant of poorer QoL as well as higher PB in caregivers of patients with liver cirrhosis. Surprisingly, alcoholic etiology of liver cirrhosis per se had no independent impact on these factors. Our findings are comparable to an Indian study conducted by Shrestha et al. [21]. Additionally, the detrimental effect of continued alcohol consumption on family members and caregivers has been studied extensively [22].

Caregivers of patients with liver cirrhosis suffer from a higher PB compared to the general population and the burden on caregivers is particularly pronounced in patients with ongoing alcohol abuse [8]. Often, there has been already a long-term burden on caregivers until the development of liver cirrhosis. A decisive therapeutic step to improve the burden of the caregivers is the optimal care of the patients. In addition to precise information about the possible clinical complications and psychotherapy may lead to an improvement in the patients' HRQoL. In other chronic diseases such as dementia, psychotherapy can lead to an improvent in patiens’ HRQoL and a reduction in caregiver burden [23]. Additionally, there is evidence that mindfulness-based stress reduction therapy also improves the HRQoL of patients with liver cirrhosis and their caregivers [24].

In our study, the severity of liver cirrhosis as reflected by MELD did not associate with poor QoL or higher PB in caregivers. However, we found an association of a history of or active HE—which is an indicator of poorer prognosis and marks the decompensated stage of liver cirrhosis—with higher PB. One explanation may be that caregivers usually do not recognize changes in MELD, since this is only a combination of laboratory values, while complications like HE may be more obvious and frightening. Nevertheless, it is an important finding that the MELD score, which is used for the assessment of the severity of liver cirrhosis and is ultimately used for organ allocation in the setting of liver transplantation, does not seem to influence QoL and PB of caregivers. These discrepancies should be taken into account in the assessment and management of patients with liver cirrhosis, especially when caregivers are involved.

In our present study, several potentially modifiable factors were identified to improve QoL or PB in caregivers. It only seems reasonable that specialized care to improve alcohol abstinence and a consequent secondary prophylaxis or even primary prophylaxis for HE may also impact caregivers’ QoL and PB. However, due to the study design, we were only able to identify associations and are therefore unable to judge the impact of these preventive strategies on an outcome like QoL. Moreover, it has to be acknowledged as a limitation of our study, that patients were enrolled at a single large German transplant center. Therefore, we cannot exclude a referral bias and our findings may not be generalizable to all patients with liver cirrhosis. Additionally, we have to acknowledge that especially our subgroup analyses of patients with or without alcoholic liver cirrhosis have to be interpreted with caution due to the comparably small sample sizes.

## Conclusion

In conclusion, we identified potentially modifiable predictors for poorer QoL as well as higher PB in caregivers of patients with liver cirrhosis. We were able to demonstrate that continued alcohol consumption was associated with poorer QoL and higher PB in caregivers, while an alcoholic etiology of liver cirrhosis per se had no impact on these factors. Additionally, we found that a history of or active HE represents a risk factor for higher PB. Moreover, regarding higher PB, caregivers’ QoL and patients’ HRQoL seem to influence each other in a negative sense. Focusing on specialized care to improve alcohol abstinence and an implementation of consequent preventive strategies for HE might improve QoL and PB in caregivers of patients with liver cirrhosis.

## Supplementary Information

**Additional file 1: Table S1.** Patient baseline characteristics stratified by etiology of liver cirrhosis at the time of study inclusion.

## Data Availability

The data supporting the conclusion of this article are includes within the article. Any queries regarding these data may be directed to the corresponding author.

## Abbreviations

CCM: Cirrhosis Center Mainz
CLDQ: Chronic liver disease questionnaire
CP: Child Pugh
HBV: Hepatitis B virus
HCV: Hepatitis C virus
HDV: Hepatitis D virus
HE: Hepatic encephalopathy
HRQoL: Health-related quality of life
IQR: Interquartile range
MELD: Model of end-stage liver disease
PB: Psychosocial burden
QoL: Quality of life
SF-36: Short Form Health 36
ZBI: Zarit Burden Interview
