# Supplementary material for: Continued alcohol consumption and hepatic encephalopathy determine quality of life and psychosocial burden of caregivers in patients with liver cirrhosis
Source: Health Qual Life Outcomes. 2022 Feb 8;20:23. doi: 10.1186/s12955-022-01923-z (PMC8822635; doi:10.1186/s12955-022-01923-z)
Supplement: Supplementary file 1 — Additional file 1: Table S1. Patient baseline characteristics stratified by etiology of liver cirrhosis at the time of study inclusion. [file 12955_2022_1923_MOESM1_ESM.docx]

**Supplementary Table 1**: **Patient baseline characteristics stratified by etiology of liver cirrhosis at the time of study inclusion.**

| Variable | Patients with alcoholic liver cirrhosis  (N = 58) | Patients with non - alcoholic liver cirrhosis  (N = 48) |
| --- | --- | --- |
| Male Gender of patients  n (%) | 34  (71%) | 38  (66%) |
| Age of patients (years)  median (IQR) | 62  (50; 68) | 64  (55; 70) |
| Age of caregiver (years)  median (IQR) | 59  (48; 66) | 59  (47; 67) |
| Sodium (mmol/l)  median (IQR) | 138  (134; 140) | 138  (136; 140) |
| Creatinine (mg/dl)  median (IQR) | 0.9  (0.8; 1.5) | 0.91  (0.8; 1.3) |
| Bilirubin (mg/dl)  median (IQR) | 2  (1.1; 3.2) | 1.5  (0.9; 1.3) |
| Albumin (g/l)  median (IQR) | 30  (25; 34) | 33  (27; 35) |
| INR  median (IQR) | 1.4  (1.2; 1.7) | 1.3  (1.1: 1.5) |
| CRP (mg/l)  median (IQR) | 9.7  (3.4; 23) | 7.4  (3.6; 12) |
| White blood cell count (/nl)  median (IQR) | 6.0  (4.7; 9) | 5.5  (4.5; 7.1) |
| Hemoglobin (g/d)  median (IQR) | 11.2  (9.5; 13) | 12.8  (10.7; 14.7) |
| Platelets (/nl)  median (IQR) | 105  (71; 139) | 98  (71; 160) |
| MELD  median (IQR) | 16  (12; 21) | 13  (8; 16) |
| Child-Pugh score  *A  n (%) | 16  (33%) | 33  (57%) |
| *B  n (%) | 22  (46%) | 19  (33%) |
| *C  n (%) | 10  (21%) | 6  (10%) |
| History of ascites  n (%) | 29  (60%) | 31  (54%) |
| History of spontaneous bacterial peritonitis  n (%) | 3  (6%) | 6  (11%) |
| History of hepatic encephalopathy  n (%) | 17  (35%) | 11  (19%) |
| History of variceal bleeding  n (%) | 9  (19%) | 12  (21%) |
| History of hepatorenal syndrome  n (%) | 7  (15%) | 4  (7%) |
